# Development of a robust TaqMan probe-based one-step multiplex RT-qPCR for simultaneous detection of SARS-CoV-2 and Influenza A/B viruses

**DOI:** 10.1186/s12866-023-03048-9

**Published:** 2023-11-11

**Authors:** Hamidreza Abbasi, Hadi Razavi Nikoo, Fatemeh Fotouhi, Ayyoob Khosravi

**Affiliations:** 1 Department of Medical Biotechnology, Faculty of Advanced Medical Technologies, Golestan University of Medical Sciences, Gorgan, Iran; 2 Department of Microbiology, Faculty of Medicine, Golestan University of Medical Sciences, Gorgan, Iran; 3 Infectious Disease Research Center, Golestan University of Medical Sciences, Gorgan, Iran; 4 Department of Influenza and other Respiratory Viruses, Pasteur Institute of Iran, Tehran, Iran; 5 Stem Cell Research Center, Golestan University of Medical Sciences, Gorgan, Iran; 6 Department of Molecular Medicine, Faculty of Advanced Medical Technologies, Golestan University of Medical Sciences, Gorgan, Iran

**Keywords:** SARS-CoV-2, Influenza a virus, Influenza B virus, Multiplex RT-qPCR, Co-infection

## Abstract

**Background:**

During the coronavirus disease 2019 (COVID-19) pandemic, the simultaneous detection of severe acute respiratory syndrome coronavirus 2 *(SARS-CoV-2*) and *Influenza A, and Influenza B* viruses is essential for rapid differential diagnosis in patients with similar symptoms, especially during “*flu* season” in the post-pandemic era. So far, several multiplex methods have been approved for the simultaneous detection of *SARS-CoV-2, Influenza A, and Influenza B*. However, due to the rapid mutation rate of the *SARS-CoV-2* genome and the emergence of new variants, existing methods must be improved and updated.

**Methods:**

To identify a highly conserved region in the *SARS-CoV-2 N-gene*, a genomic survey was performed to increase the sensitivity and specificity of primer and probe sets targeting the *SARS-CoV-2* genome. The 95% LLOD (95% lower limits of detection) were calculated by probit analysis. A total of 70 predetermined clinical samples using singleplex RT-qPCR assays, were included. The clinical performance of the multiplex RT-qPCR assay was determined and compared with a commercial multiplex kit. The Cohen’s kappa coefficient, *P*-value (McNemar’s test), Passing-Bablok regression, and Bland Altman agreement analysis were determined to monitor the agreement of the assays.

**Results:**

The novel *SARS-CoV-2* primer and probe set designed in this assay was able to detect all variants of concern (VOCs) and variants of interest (VOIs) with high analytical and clinical performance. The 95% LLOD for the multiplex RT-qPCR was 20 copies per reaction for the *N* gene of *SARS-CoV-2*, 2 copies per reaction for *M1* gene of *Influenza A* and *NS1* gene of *Influenza B*. The diagnostic sensitivity of the multiplex RT-qPCR was 94.4%, 93.7%, and 100% for the detection of *SARS-CoV-2*, *Influenza A*, and *Influenza B* genomes, respectively. Moreover, the specificity was identical (100%) in both assays. According to the agreement analysis results, there was no statistical difference between our multiplex assay and the commercial kit.

**Conclusions:**

In this study, we developed a novel in-house made multiplex RT-qPCR assay, with high sensitivity, specificity, and reliability for the diagnosis of *SARS-CoV-2* infection in clinical samples. This is valuable during *Influenza* seasons when influenza co-circulates with SARS-CoV-2, as it saves costs, time, and thus specific and timely treatment of patients.

**Supplementary Information:**

The online version contains supplementary material available at 10.1186/s12866-023-03048-9.

## Introduction

*SARS-CoV-2*, *Influenza A, and Influenza B* are significant global health threats with the potential of causing economic, medical, and severe public health crises with millions of infections and deaths throughout the world. These viruses are the most important human pathogens and usually cause mild upper respiratory diseases that spread rapidly among people [1–5]. We now critically needed an accurate and readily available molecular diagnostic technique to screen and identify patients infected with these pathogens. As a significant trait that influences their infectivity and COVID-19 pandemic patterns, *Influenza* viruses have been known to be important causative agents of co-infection in patients, which rise mainly in the *flu* season. Viral co-infections with COVID-19 disease are known as a major cause of morbidity and mortality and can pose a challenge to healthcare providers because they have very similar clinical features [6–9]. However, individuals infected with *SARS-CoV-2* have a longer incubation period (2–14 days) until the onset of clinical symptoms compared to individuals infected with *influenza A* and *B* [10]. In light of this fact, the design and development of an in-house made one-step multiplex RT-qPCR can help accurately diagnose and differentiate *SARS-CoV-2* from other respiratory viruses to manage COVID-19 patients and reduce time and cost. Multiplex RT-qPCR is considered one of the best methods for the detection of *SARS-CoV-2* and *Influenza* viruses in clinical samples using multiple primer and probe sets that bind different targets to specifically amplify it [11–13]. In this study, we aim to develop a robust method for a cheap and time-saving procedure to detect and differentiate *SARS-CoV-2* and *Influenza A and B*, and consequently, specific and timely treatment of patients, and also highlighted the pitfalls of the one-step multiplex RT-qPCR assay. For this purpose, *SARS-CoV-2* genomic survey was performed to increase the sensitivity and specificity of the primer and probe binding to the *SARS-CoV-2* genome.

## Methods

### In-silico studies for primer and probe set design

**Fig. 1:**
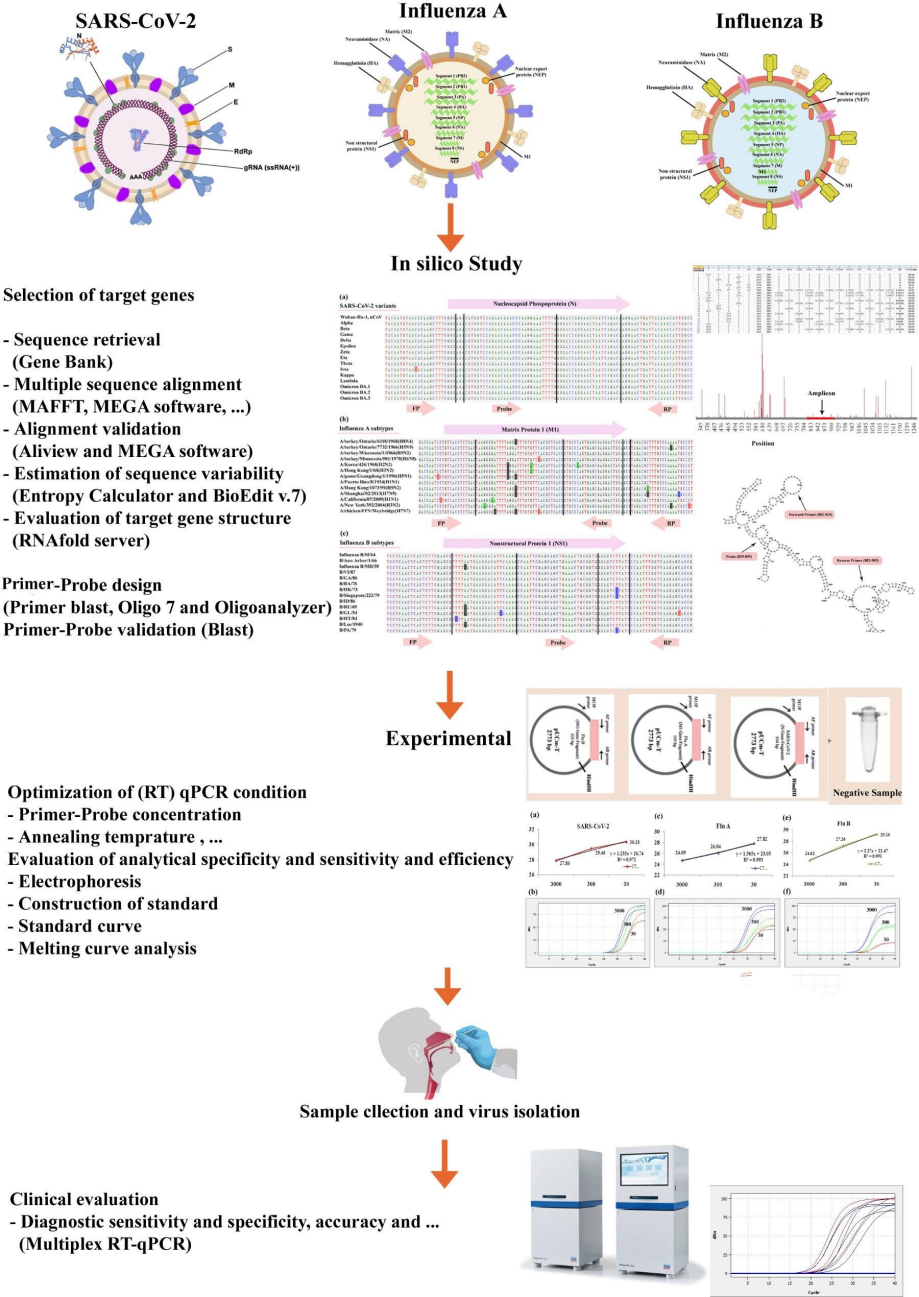
A simple workflow for designing and developing of the multiplex assay. For these purposes, (1) in silico approaches, such as gene target selection, multiple alignments, sequences processing and primer-probe sets design, and (2) experimental methods, such as real time PCR optimization, and analytical and clinical evaluation of the assays are crucial

Nucleotide sequences of the *SARS-CoV-2 N*-gene (~ 43,000 sequences) were obtained from the NCBI database (https://www.ncbi.nlm.nih.gov/sars-cov-2/, accessed on March 2022) and aligned to identify conserved and non-conserved regions. Usegalaxy server (https://usegalaxy.org), Aliview software version 1.26, and MEGA10 software were used for sequence editing and minor manual adjustments. The selected sequences included *Omicron BA.3* (78 sequences), *Theta* (42 sequences), and the *Wuhan* reference sequence (40,000 sequences). There were 200 sequences for variants including *Alpha*, *Beta*, *Gamma*, *Delta*, *Omicron BA.1*, *Omicron BA.2*, *Omicron BA.4*, *Omicron BA.5*, *Epsilon*, *Zeta*, *Eta*, *Iota*, *Kappa*, and *Lambda* (Table 1). To gain insight into the alignment heterogeneity, we calculated the positional nucleotide numerical summary (PNNS) and entropy values (H*(i)*) by using the Alignment Explorer server (http://www1.szu.cz:8080/EntropyCalcWeb). The entropy plot was made by using Microsoft Excel. Specific primer and probe set for the detection of *SARS-CoV-*2, *Influenza A*, and *Influenza B* are shown in Table 2. When designing and selecting multiple PCR primer and probe sets for multiplex assay, it is important to consider the compatibility between primer and probe sets, such as dimerization of oligonucleotides or secondary structures, GC content, primer and probe sets Tm, amplicon length, and oligonucleotides specificity. The IDT OligoAnalyzer™ tool (https://www.idtdna.com/pages/tools/oligoanalyzer), Oligo (version 7.60), and AlleleID 6 software were used for oligonucleotides analysis. The presence of secondary structures in primer and probe binding sites can cause poor amplification efficiency; therefore we designed the primer and probe to hybridize to the cyclic structure. Secondary structure and primer-probe binding sites of the *N* gene predicted by the RNAfold web server (http://rna.tbi.univie.ac.at/cgi-bin/RNAWebSuite/RNAfold.cgi). We also aligned the *N*-gene of *SARS-CoV-2* with other coronaviruses such as *SARS-CoV-1, MERS-CoV, HCoV-229E, HCoV-NL63, HCoV-HKU1*, and *HCoV-OC43* to identify conserved and non-conserved regions among coronaviruses. A simple workflow for the design and development of our multiplex assay is shown in Fig. 1.

**Table 1:** Selected sequences related to variants of *SARS-CoV-2* for multiple alignments

Variants	Lineage	Synonyms	Origin /Date	Selected Sequences
Reference	19 A	Wuhan-Hu-1, nCoV	China/ Dec 2019	40,000
Variant of concern (VOC)	Alpha	B.1.1.7	United Kingdom/ December 2020	200
	Beta	B.1.351, B.1.351.2, B.1.351.3	South Africa/ December 2020	200
	Gama	P.1, B.1.1.28.1, P.1.1,P.1.2	Brazil/ January 2021	200
	Delta	B.1.617.2, AY.1, 2	India/ May 2021	200
	Omicron BA.1	BA.1	South Africa/ Dec 2021	200
	Omicron BA.2	BA.2	South Africa/ Dec 2021	200
	Omicron BA.3	BA.3	South Africa/ Dec 2021	78
	Omicron BA.4	BA.4	South Africa, Jan 2022	200
	Omicron BA.5	BA.5	South Africa, Jan 2022	200
Variant of Interest (VOI)	Epsilon	B.1.427, B.1.429	California/ July 2020	200
	Zeta	P.2, B.1.1.28.2	Brazil/ Oct 2020	200
	Eta	B.1.525,	United Kingdom/Nigeria December 2020	200
	Theta	P.3, B.1.1.28.3	Philippines/ January 2021	42
	Iota	B.1.526, 21 F	United States/ November 2020	200
	Kappa	B.1.617.1	India/ December 2020	200
	Lambda	C.37, B.1.1.1.C37	Peru/ August 2020	200

**Table 2:** Primers and probes used in our multiplex RT-qPCR

Assay Primer- probe	Target gene	Oligonucleotide Sequence (5’-3’)	Tm (℃)*	Amplicon size (nt)	Concentration (µM)	Ref.
S2V FP RP Probe	N	TACAATGTAACACAACCTTTCGGC GACCTATGTTTGTAATCAGTTCCT TAMRA-CGTGGTACAGAACAAACCCAAGGTAATTTTG-BHQ2	63.7 60.7 67.8	104	0.225 0.225 0.8	This study
IAV FP RP Probe	M1	GACCRATCCTGTCACCTCTGAC AGGGCATTYTGGACAAAKCGTCTA FAM-TGCAGTCCTCGCTCACTGGGCACG-BHQ1	64 66 73	109	0.15 0.15 0.15	[31]
IBV FP RP Probe	NS1	TCCTCAAYTCACTCTTCGAGCG CGGTGCTCTTGACCAAATTGG JOE-CCAATTCGAGCAGCTGAAACTGCGGTG-BHQ1	64 63.8 70.7	103	0.15 0.15 0.15	[32]
IC FP RP Probe	RNase P	AGATTTGGACCTGCGAGCG GAGCGGCTGTCTCCACAAGT ROX-TTCTGACCTGAAGGCTCTGCGCG-BHQ2	64.4 66.2 70	65	0.225 0.225 0.5	[11]

*Tm calculated with OligoAnalyzer Tool

### Construction of positive control plasmids

The *SARS-CoV-2 N*, *Influenza A M1*, and *Influenza B NS1* amplicons were amplified by RT-PCR from positive clinical samples. Amplicons were cloned into the pUCM-T vector using the BioBasic TA Cloning kit (BioBasic, Canada) according to the manufacturer’s instructions to obtain the appropriate plasmids. Purified plasmids were sequenced to confirm sequence accuracy (Supplemental Information 1) and were also used to test for their sensitivity and specificity in the conventional PCR and to derive standard curves for the multiplex RT-qPCR assay. Copy number (copies/µL) was calculated using the following equation: [C (ng/µL)*6.022 × 10^23^**/** N*660 (g/mol)*10^9^ (ng/g)], in which C represents the concentration of plasmid (ng/µL); N is the length of the plasmid (number of nucleotides), and 660 is the average mass of 1 bp dsDNA [14].

### SYBR Green real-time PCR

When designing multiplex RT-qPCR assays, it is critical to choose primers that are highly specific and do not produce primer dimers. Before multiplexing, all primer sets were tested under singleplex, duplex, triplex and quadruplex conditions using the SYBR Green RT-qPCR assay. The SYBR Green RT-qPCR assay was performed using the *ABI* StepOnePlus™ Real-Time PCR System (Applied-Biosystems) in the presence of 2X SYBR-Green PCR Master Mix (Sinacolon, Iran). We carefully optimized the real-time PCR conditions to minimize primer dimer formation by optimizing the primer concentration and the temperature using the melting curve analysis. Amplifications were performed in a final volume of 20µL, including 5µL of positive control plasmids as a template. Cycling conditions were optimized according to the amplicon size and the Tm of primers, ending with a melting curve from 60 to 90 °C. Fluorescence was measured at the end of each cycle. After optimization, we performed SYBR Green real-time PCR amplification using the quantified *SARS-CoV-2* positive control plasmid. Standard curves were generated by plotting Cycle threshold (Ct) versus plasmid copy number. Linear regression analysis was carried out for the *SARS-CoV-2 N* gene.

### TaqMan probe-based multiplex RT-qPCR assay condition

Multiplex RT-qPCR was optimized for all reactions and performed using the one-step RT-qPCR Master Mix (Pishtazteb co, Tehran, Iran), in *ABI* StepOnePlus™ Real-Time PCR System (Applied-Biosystems). The reaction mixture (final volume: 20 µL) consists of 9 µL of RT-qPCR solution, 0.225 µM of each *SARS-CoV-2* and *Rnase P* forward and reverse primers, 0.8 µM of the *SARS-CoV-2* probe, 0.15 µM of each *Influenza A and Influenza B* primers and probes sets, 0.5 µM of the probe for *RNase P* and 10µL of RNA template. *The RNase P* gene was used as an internal control for RNA extraction, sampling, and RT-qPCR process monitoring to avoid false-negative results. To monitor cross-dimer or contamination, we added nuclease-free water to negative control tubes. Thermal cycling conditions were performed as follows: 20 min at 50 °C for reverse transcription, 3 min at 95 °C for PCR initiation activation, and 40 cycles of 95 °C for 10s (denaturation) and 55 °C for 35s (annealing/extension).

### LLOD (lower limits of detection) of the multiplex RT-qPCR assay

After optimizing primer and probe sets under different conditions (singleplex to multiplex) using positive control plasmids, we generated standard curves for all target genes. After diluting the plasmids in a 10-fold ratio (ranging from 3 × 10^5^, 3 × 10^4^, 3 × 10^3^, 3 × 10^2^, 3 × 10^1^, 3 × 10^0^, and 0.3 copies per reaction), standard curves were generated by plotting Cycle threshold (Ct) versus plasmid copy number and linear regression analysis were carried out for the *N*, *M1*, and *NS1* gene targets. The analytical sensitivity of the multiplex RT-qPCR assay was measured by testing 10-fold serial dilutions of quantified standard plasmids containing *SARS-CoV-2*, *Influenza A*, and *Influenza B* gene fragments. Each dilution was tested in triplicate by the multiplex RT-qPCR assay. The 95% lower limits of detection (95% LLOD) were calculated using probit analysis. For the determination of 95% LLOD, each dilution was tested in 6 replicates on two independent runs, and the lower limit of detection (LLOD) was defined as the concentration of copies/reaction of the lowest dilution that could be detected with 95% probability.

### The precision of the multiplex RT-qPCR assay

Repeatability (intra-assay precision) and reproducibility (inter-assay precision) of the multiplex RT-qPCR assay were determined using three different concentrations (5 × 10^5^, 5 × 10^3^ and 5 × 10^1^ copies per reaction) of each plasmid standard. For intra-assay, each dilution was analyzed in triplicate in one reaction, while for inter-assay; each dilution was analyzed in three independent reactions on day 1, day 2, and day 3. Intra- and inter-assay were calculated for each dilution and expressed as % coefficients of variation (%CV).

### Analytical specificity

We evaluated the analytical specificity of the multiplex assay by using common respiratory pathogens, including *Influenza A* virus, *Influenza B* virus, *SARS-CoV-2*, *Respiratory Syncytial Viru*s (*A* and *B*), *Rhinovirus*, *Adenovirus* (*B* and *C*) and *Epstein-Barr virus* (*EBV*).

### Evaluation of clinical performance

For evaluation of the clinical performance, a total of 70 archived predetermined respiratory swab specimens were subjected to the multiplex RT-qPCR assay. The samples were previously tested for *SARS-CoV-2*, *Influenza A*, and *Influenza B* by singleplex RT-qPCR diagnostic assays as the gold standard. The singleplex RT-qPCR tests were performed using the one-step RT-qPCR kit (Pishtaz Teb Diagnostics, Tehran, Iran), according to the manufacturer’s instructions. To validate the outcome of the multiplex RT-qPCR assay, we compared our results and tested the clinical samples with Viga *SARS-CoV-2* and *Influenza A/B* molecular diagnostic kit (ROJE Technologies, Iran). Out of these samples, 18 were positive for *SARS-CoV-2*, 16 were positive for *influenza A*, 10 were positive for *influenza B*, and 26 were negative for *SARS-CoV-2* and *influenza A* and *B*. The specimens were placed into sterile tubes containing 3ml of viral transport media (VTM) consisting of Hank’s balanced salt solution at pH 7.4 containing BSA (1%), amphotericin (15 µg/mL), penicillin G (100 units/mL), and streptomycin (50 µg/mL). Viral RNA was extracted from 200 µL of the VTM by using an RNA extraction kit (RNJia kit, ROJE Technologies, Iran), and eluted in 60 µL of nuclease-free water. RNA extracts were stored at -70 °C for further analyses. The agreement between our multiplex assay and the commercial multiplex kit was evaluated using Cohen’s kappa index, McNemar’s test (*P*-value), Passing-Bablok regression, and Bland Altman agreement analysis. Passing-Bablok regression was used to compare correlations Ct values in clinical samples between our multiplex assay and commercial multiplex kit. Bland-Altman analysis was used to determine the bias and limits of agreement between the two assays, corresponding to the 95% CI of the mean bias of all paired measurements.

### Testing of co-infections

To evaluate the performance of the current assay in cases with co-infections, we included and combined different viruses (containing of: *SARS-CoV-*2, *Influenza A* and *Influenza B*) with different Ct values. We prepared a total of 8 co-infected samples for *SARS-CoV-2* and *Influenza A* and *B* viruses.

### Statistical analysis

Statistical analysis including mean, standard deviation (SD), and coefficient of variation (CV %) for Ct values, box plot, and creating of entropy plot were performed using Microsoft Excel. Statistical analysis, including Probit regression analysis (at the 95% probability level to determine the detection limits), Passing-Bablok regression, and Bland Altman agreement were performed with MedCalc Statistical Software version 22.007 (MedCalc Software Ltd, Ostend, Belgium; https://www.medcalc.org). McNemar’s test and the kappa index were used to analyze the statistical difference and agreement between the methods by SPSS Version 22 (IBM Corp., USA). A *P*-value of < 0.05 was considered statistically significant.

## Results

### Primer and probe sets were specific to *SARS-CoV-2*, *Influenza A, and Influenza B*

**Fig. 2:**
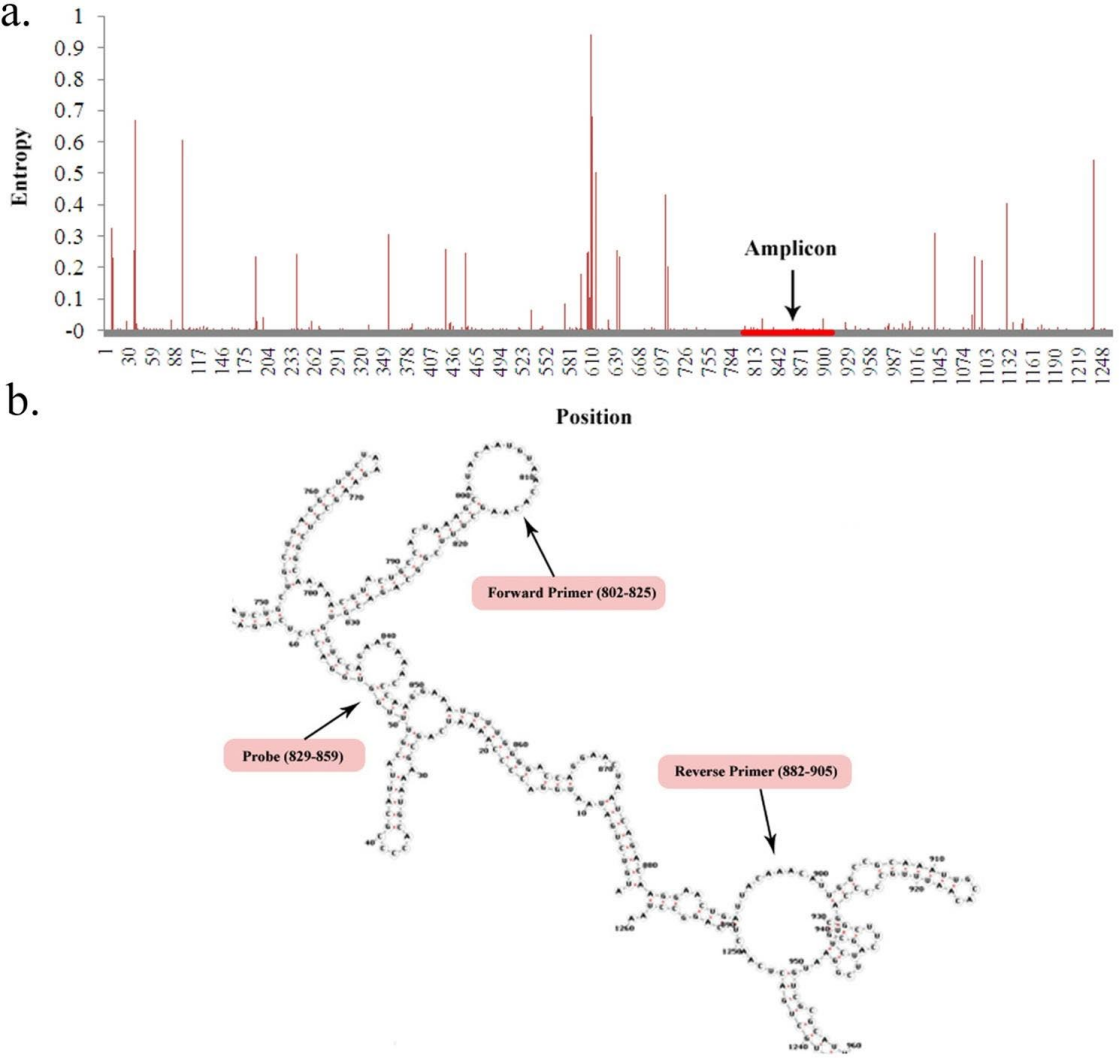
The variability analysis of aligned sequences and prediction of secondary structure of the *N* primers and probe annealing sites. After “Positional nucleotide numerical summary calculation” the entropy values were calculated for the entire of the *N* gene. The entropy plot was obtained by plotting the entropy values against the *N* gene positions. The variation per position is expressed by the column height. The conserved region as amplicon is shown with red line (**a**). The primers and probe designed to hybridized with cyclic structure (no stem structure) (**b**)

In the first of the COVID-19 pandemic, all nucleotide sequences of the *SARS-CoV-2* were obtained from GenBank that belong Wuhan strain. During the waves of the pandemic and the continuously evolving nature of *SARS-CoV-2*, we added available nucleotide sequences of the variants (*Alpha, Beta, Gama, Delta, Omicron BA.1, Omicron BA.2, Omicron BA.2.12.1, Omicron BA.4, Omicron BA.5*). The variability analysis of *N* gene sequences using positional nucleotide numerical summary calculation (PNNS) and entropy plot (Fig. 2a) shows that a highly conserved region (802–905 bp) was selected as *SARS-CoV-2* primer and probe annealing site. The compatibility analysis of primer and probe sets by in-silico approaches (IDT OligoAnalyzer™ and BLAST tools) showed some degree of homo- or hetero-dimerization. Therefore, we created primer/probe-template mismatches to reduce the possibility of cross-dimer formation between primer and probe sets. The mismatches are located at the 5′ ends of the *N* gene primers and probe.

**Fig. 3:**
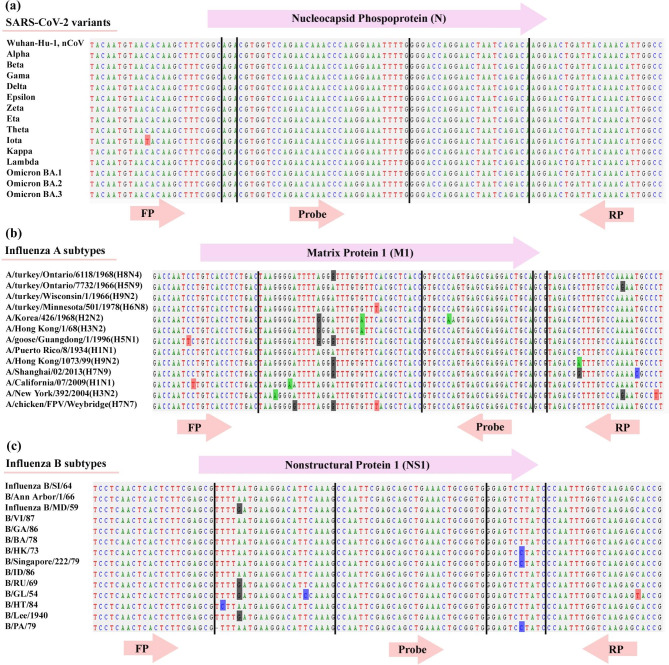
Alignment of *SARS-CoV-2*, *Influenza A* and *Influenza B* primer and probe sets with consensus sequences of *SARS-CoV-2*, *Influenza A*, and *Influenza B* subtypes

The secondary structure of the *N* gene and the primer and probe binding sites are shown in Fig. 2b. Multiple sequence alignment confirmed that our proposed assay is specific for all subtypes of *SARS-CoV-2*, *Influenza A*, and *Influenza B*; no evidence of non–*SARS-CoV-2*, non- *Influenza A*, and *non-Influenza B* target matches was found (Fig. 3).

The multiple sequence alignment of *SARS-CoV-2 N*-gene with other human coronaviruses showed that primer and probe binding sites were conserved among the *SARS-CoV-2* variants, but not among other human coronaviruses (Supplemental information 2).

### Optimization primer sets using SYBR Green Real-time PCR

Before optimization and creation of mismatches in *SARS-CoV-2-N* primers, electrophoresis and melting curve analysis of the amplified *N* gene using conventional RT-PCR and SYBR Green real-time PCR showed the presence of a primer dimer (Supplemental information 3 (a)). After creating intentional mismatches and optimizing primer concentrations and cycling conditions under singleplex, duplex, triplex, and quadruplex conditions using SYBR Green real time-PCR, the primer dimer formation was mitigated. The melting curves obtained for all conditions are shown in supplemental information 3 (b-f). The curves showed only one peak corresponding to the amplification product, resulting in no primer dimmers. We also performed SYBR Green real-time PCR with *N* gene primers in duplicate using copy numbers of 3 × 10^6^, 3 × 10^5^, 3 × 10^4^, 3 × 10^3^, 3 × 10^2^ and 30 per reaction. The amplification plots, standard curve, and R^2^ score are summarized in supplemental information 3 (g). A linear regression relationship was observed with an R^2^ score of 0.99 for the *SARS-CoV-2 N* gene.

### The multiplex assay has a high analytical sensitivity

The results showed that the optimized SYBR Green real time-PCR using *SARS-CoV-2-N* primers under singleplex conditions can detect 30 copies per reaction of positive control plasmid. The analytical sensitivity of the singleplex assays for *Influenza A* and *Influenza B* was determined to be 5 copies per reaction [31 and 32].

**Fig. 4:**
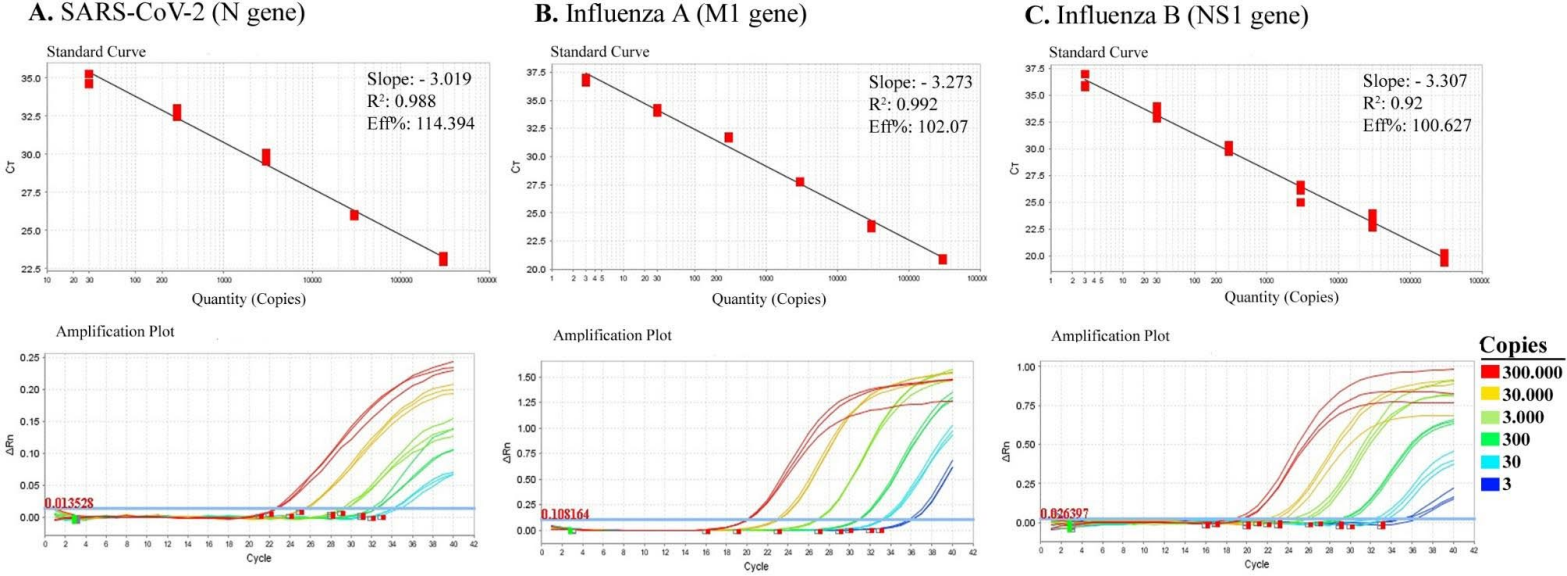
The standard curves and amplification plots of our multiplex assay. A 10- fold serial dilution of plasmid standards containing cloned target sequences was prepared. The standard curves and amplification plots of the *SARS-CoV-2-N* gene (**A**), standard curves and amplification plots of the *Influenza A-M1* gene (**B**) and standard curves and amplification plots of the *Influenza B-NS1* gene (**C**)

The obtained standard curves (E, slope, and R^2^ score) and amplification plots for the *N*, *M1*, and *NS1* genes using the multiplex assay are summarized in Fig. 4. The R^2^ scores were determined as 0.988 for the *N*, 0.992 for *M1*, and 0.920 for the *NS1* gene. The analytical sensitivity of the multiplex assay on standard plasmids were 30 copies for the *SARS-CoV-2*, and 3 copies for both *Influenza A* and *B*, which corresponds to averaged Ct values of 34.84, 36.95, and 36.21 for *N*, *M1*, and *NS1* genes, respectively (Supplemental information 4). These results were also in agreement with the singleplex real time-PCR assay. The 95% LLOD was determined for each gene in our multiplex RT-qPCR assay by probit analysis using positive control plasmid dilutions. The 95% LLOD was calculated to be 20 copies per reaction for the *N* gene and 2 copies per reaction for the *M1* and *NS1* genes.

### Intra- and inter-assay variability

The results of intra- and inter-assays (Table 3) for the multiplex detection of *N, M1, and NS1* genes revealed that the coefficient of variation (CV%) was all < 5%, which suggested the multiplex RT-qPCR assay is an accurate and reliable diagnostic tool for detection of the viruses.

**Table 3:** Intra- and Inter-assay in multiplex detection of *SARS-CoV-2* (*N* gene), *Influenza A* (*M1* gene), and *Influenza B* (*NS1* gene)

Copies of plasmid	Gene	Ct value in Intra-Assay			Mean ± SD	CV%	Ct value in Inter-Assay			Mean ± SD	CV%
		1	2	3			Day1	Day2	Day3		
5 × 10^5^	N	20.75	21.08	21.56	21.13 ± 0.40	1.93	20.27	20.58	21.68	20.84 ± 0.74	3.55
	M1	19.89	19.30	19.30	19.49 ± 0.33	1.74	19.59	20.01	19.80	19.80 ± 0.21	1.06
	NS1	18.47	18.46	18.41	18.45 ± 0.03	0.17	18.42	19.22	19.04	18.89 ± 0.42	2.22
5 × 10^3^	N	26.49	25.43	26.63	26.18 ± 0.65	2.51	26.94	27.62	27.33	27.30 ± 0.34	1.25
	M1	26.97	26.57	27.92	27.15 ± 0.69	2.55	25.45	26.42	26.28	26.05 ± 0.52	2.00
	NS1	25.13	25.13	25.02	25.10 ± 0.06	0.24	26.81	25.93	26.84	26.53 ± 0.51	1.94
5 × 10^1^	N	33.68	33.77	33.12	33.52 ± 0.35	1.05	33.77	35.71	34.81	34.76 ± 0.97	2.79
	M1	33.52	31.00	33.09	32.54 ± 1.35	4.15	33.54	33.50	32.72	33.25 ± 0.46	1.39
	NS1	31.12	32.39	31.58	31.69 ± 0.64	2.03	32.20	33.10	32.70	32.67 ± 0.45	1.38

Ct, Cycle threshold; SD, Standard Deviation; CV, Coefficient of Variation

### The primer and probe sets had high analytical specificity among other respiratory viruses

The specificity tests showed that no amplification signals were detected using our multiplex assay for all four respiratory pathogens including *respiratory syncytial virus* (*RSV*), *rhinovirus*, *adenovirus*, and *Epstein-Barr virus* (*EBV*). We also observed no cross-reactivity among the three targets (*SARS-CoV-2* and *influenza A* and *B*) within the multiplex assays (Supplemental information 5). These results suggest that our multiplex assay has good specificity for all target genes (*N*, *M1*, and *NS1*). The respiratory pathogens were isolated from clinical specimens and collected from subjects tested for these pathogens. In this study, mean Ct values for *respiratory syncytial virus* (*RSV*), *rhinovirus*, *adenovirus*, and *Epstein-Barr virus* (*EBV*) were, 21.53 ± 3.65, 22 ± 3.20, 24 ± 2.25, and 28 ± 2.25 respectively.

### Our assay showed satisfactory clinical performance and agreement

**Fig. 5:**
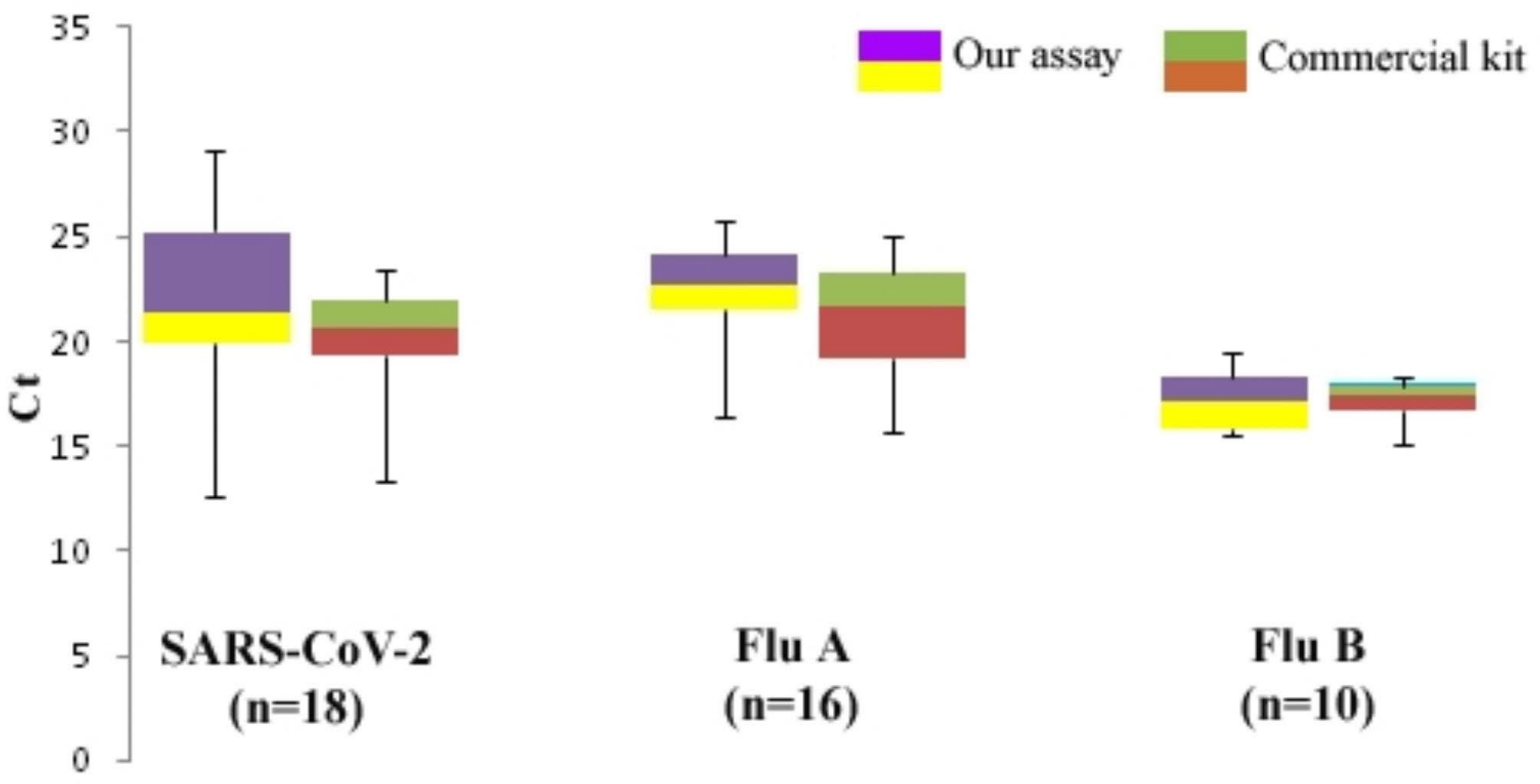
Comparative analysis of Ct values between our multiplex assay and commercial multiplex kit using clinical samples. The average Ct value of the samples detected by our multiplex assay is slightly higher than the commercial multiplex kit. However, the results indicate that our primer and probe sets are favorable for simultaneous detection of *SARS-CoV-2*, *Influenza A* and *Influenza B* viruses

The clinical performance of our proposed assay confirmed the high level of diagnostic sensitivity, specificity, accuracy, and agreement with reference singleplex RT-qPCR assays. Of the 18 *SARS-CoV-2* samples, one tested negative, and of the 16 *Influenza A* samples, one tested negative by our multiplex assay. In contrast, of the 18 *SARS-CoV-2* samples, two tested negative, and of the 16 *Influenza A* samples, one tested negative by the commercial multiplex kit. All 10 previously-tested *Influenza B* positive samples and 26 previously-tested negative samples were confirmed by both assays (Table 4) It should be noted that two clinical samples that were considered positive by the reference singleplex RT-qPCR assays were detected as negative by both multiplex methods. The samples with a Ct value of ≥ 37 are considered negative. The amplification curves of the clinical samples obtained by our multiplex assay are shown in supplemental information 6. A comparison of the Ct values ​​of each assay for the *N*, *M1*, and *NS1* genes is shown in Fig. 5. A comparison of the various parameters of the multiplex assay and the commercial kit is provided in the supplemental information 7.

**Table 4:** Comparison of our multiplex assay results versus with commercial multiplex kit in clinical samples

Virus	Clinical samples confirmed by singleplex RT-qPCR assay		Agreement					
			Commercial multiplex kit			Our multiplex assay		
	Number		Number		Ct mean	Number		Ct mean
	Positive	Negative	Positive	Negative		positive	Negative	
SARS-CoV-2	18	26	16	26	20.34	17	26	21.62
IAV	16	26	15	26	21.19	15	26	22.56
IBV	10	26	10	26	17.13	10	26	17.16
Total	44	26	41	26	19.55	42	26	20.44

IAV, Influenza A Virus; IBV, Influenza B Virus

Using the clinical specimens previously tested by singleplex RT-qPCR assays, the sensitivity, specificity, accuracy, positive predictive values, and negative predictive values of our multiplex assay and the commercial multiplex kit were calculated (Table 5). The multiplex assay detected *SARS-CoV-2, Influenza A*, and *Influenza B* with sensitivity of 94.4%, 93.7%, and 100%, respectively. The sensitivity level of the commercial kit in detecting *SARS-CoV-2, Influenza A*, and *Influenza B* was 88.8%, 93.7%, and 100%, respectively. The assay specificity for our multiplex assay and the commercial multiplex kit was 100%. The accuracy of the multiplex assay in detecting *SARS-CoV-2*, *Influenza A*, and *Influenza B* was 97.7%, 97.7%, and 100%, respectively, while the accuracy of the commercial kit was 95.4%, 97.7%, and 100%.

**Table 5:** Summary of clinical performance of our multiplex assay and commercial multiplex kit compared to reference simplex assays

Virus (target)	Our Multiplex assay				Sensitivity TP/TP + FN	Specificity TN/FP + TN	Accuracy TP + TN/TP+ TN + FP + FN	Positive predictive values TP/TP + FP	Negative predictive values TN/TN + FN	Kappa index	*P*-value (McNemar Test)
	TP	TN	FN	FP							
SARS CoV-2	17	26	1	0	0.944	1	0.977	1	0.962	0.953	1.00
IAV	15	26	1	0	0.937	1	0.977	1	0.962	0.949	1.00
IBV	10	26	0	0	1	1	1	1	1	1.000	1.00
Total	42	26	2	0	0.954	1	0.971	1	0.928	0.940	0.50
	**Commercial kit**										
SARS CoV-2	16	26	2	0	0.888	1	0.954	1	0.928	0.904	0.50
IAV	15	26	1	0	0.937	1	0.977	1	0.962	0.949	1.00
IBV	10	26	0	0	1	1	1	1	1	1.000	1.00
Total	41	26	3	0	0.931	1	0.957	1	0.896	0.910	0.25

TP, True Positive; TN, True Negative; FN, False Negative; FP, False Positive; IAV, Influenza A Virus; IBV, Influenza B Virus

The agreement analysis results showed that there was no statistical difference between our multiplex assay and the commercial multiplex kit (McNemar’s test, *P*-value ˃ 0.05). Also, the Cohen’s kappa coefficient calculated in our multiplex assay with singleplex RT-qPCR assay, and the commercial kit with singleplex RT-qPCR assay was more than 94% and 90% respectively. So our multiplex had more agreements in detecting *SARS-CoV-2*, *Influenza A*, and *Influenza B* (Table 5).

**Fig. 6:**
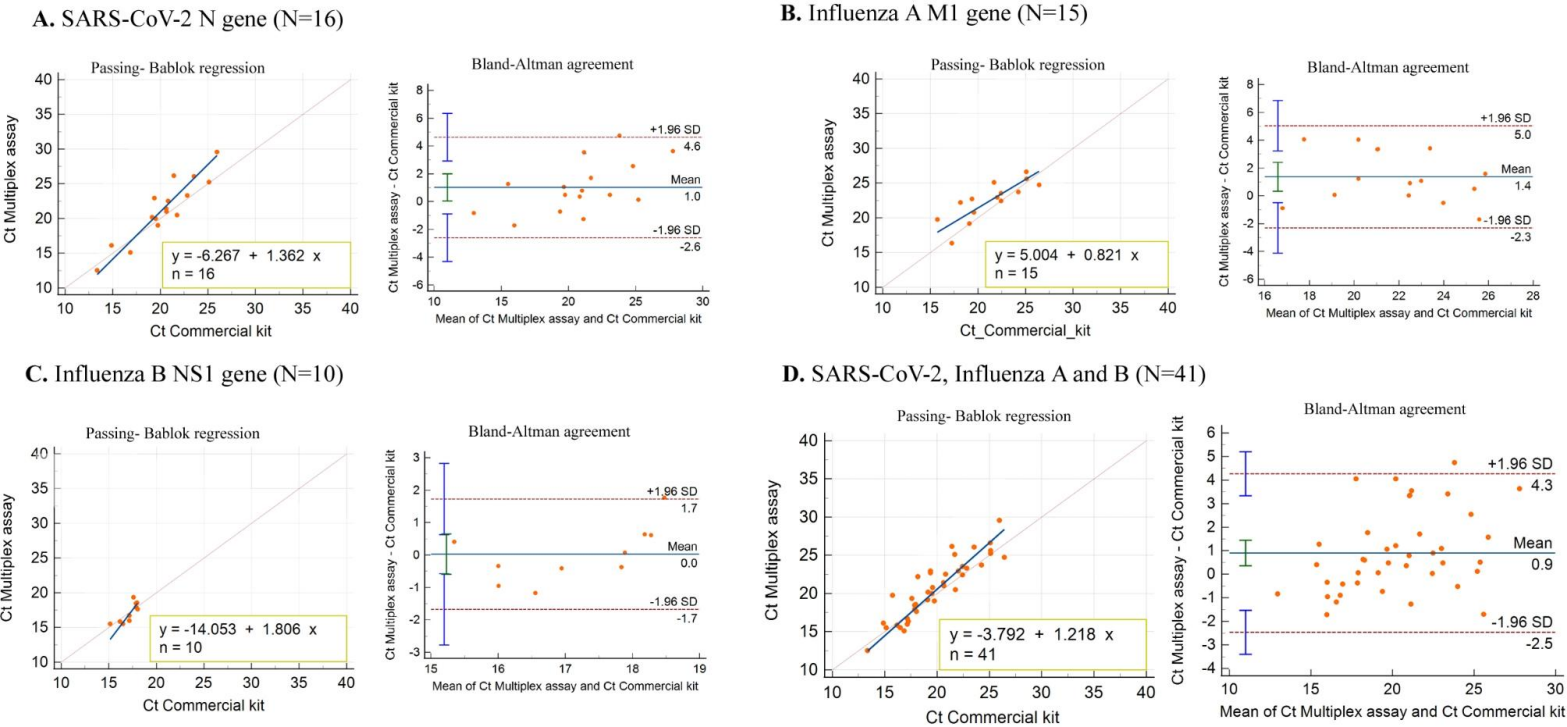
Summary of Passing-Bablok regression and Bland-Altman plots of Ct values obtained by our multiplex assay and commercial multiplex kit. Passing-Bablok regression and Bland-Altman plots for *SARS-CoV-2 N* gene (**A**), *Influenza A M1* gene (**B**), *Influenza B NS1* gene (**C**), and all genes (**D**). For Passing-Bablok regression plots, the dashed line indicates the ideal line, whereas the solid line shows the regression line of the distribution. For the Bland-Altman analyses, the solid line indicates the mean relative difference, and the dotted lines show the superior and inferior limits of agreement. Ct, cycle threshold

The Passing-Bablok regression analyses between the Ct values of positive samples for *SARS-CoV-2*, *Influenza A*, and *Influenza B* obtained by both the multiplex assay and the commercial multiplex kit are shown in Fig. 6. There was a high correlation between Ct values obtained by the two assays for *SARS-CoV-2* (R^2^ = 0.872 for the *N* gene), *Influenza A* (R^2^ = 0.874 for the *M1* gene), and *Influenza B* (R^2^ = 0.827 for the *NS1* gene). The Bland-Altman plots of between the Ct values obtained by the two assays on positive samples along with mean bias and their limits of agreement are shown in Fig. 6.

### All the co-infected samples were detected using our multiplex assay

The results of the co-infected testing indicated that our multiplex assay can detect all target genes simultaneously. Calculated Ct values and amplification plots for 8 co-infected samples by our multiplex assay and commercial multiplex RT-qPCR kit are shown in supplemental information 8 and 9.

## Discussion

The high prevalence of *SARS-CoV-2*, *Influenza A*, and *Influenza B* imposes a high financial burden on the healthcare system with millions of infections and deaths worldwide [15]. We now critically needed a fast, accurate and easily accessible diagnostic technique. The RT-qPCR assay allows amplification of nucleic acids using a primer and probe that bind to specific regions of the target viral genome to increase the assay specificity [10]. The multiplex RT-qPCR differs from the RT-qPCR in that it requires more than one set of primer and probe, which reduces the overall cost and time of the RT-qPCR assay [16]. Another advantage of multiplex RT-qPCR is the diagnosis and accurate differentiation of *SARS-CoV-2* from other respiratory viruses such as *Influenza A* and *B* viruses, which manage COVID-19 patients with co-infections and secondary infections [17, 18]. Thus, the design and development of an in-house made one-step multiplex RT-qPCR method can help slow or stop the spread of these viruses. Two points were considered in the primer and probe sets design based on the genome of *SARS-CoV-2* variants. The first was to identify the conserved and the unique region of the *N*-gene with the minimum entropy. The second was to recognize the non-stem structure of the *N*-gene, which can be easily accessed using primers and probe.

Researchers have designed RT-qPCR primer and probe for various targets in the *SARS-CoV-2* genome, including the *S, N*, *E*, and *RdRp* genes [11, 19]. Our previous study shows that the *N* gene has higher specificity compared to the other *SARS-CoV-2* genes and is also a better target for identifying new cases of *SARS-CoV-2* in clinical samples, based on the real-time PCR Ct value. The multiplex RT-qPCR assay for simultaneous detection and differentiation of *SARS-CoV-2* and other respiratory viruses has limitations related to the number of used primer and probe sets. Hence, we must use one set of primer and probe for *SARS-CoV-2* detection [11]. In addition, the occurrence of mutations in the *S, N*, *E*, and *RdRp* genes is high, which can lead to primer and probe mismatches at annealing sites and increase false-negative result, especially for new variants with many mutations. Evaluation of the *SARS-CoV-2* primer and probe sets of recommended by WHO and other studies showed that many mutations were located in annealing sites of most primers and probes [20–23].

Overall, a 104 bp region of the *SARS-CoV-2 N*-gene was identified using the RNAfold Web Server, which at 60 °C (RT-qPCR reaction temperature) had the lowest secondary structure and entropy compared to other regions of the genome (Fig. 2a-b). We also design for the first time a high coverage primer and probe set that were conserved among the *SARS-CoV-2* variants as shown in Figs. 2 and 3, but were not conserved among other human coronaviruses.

The sensitivity of the multiplex RT-qPCR for detection of the *SARS-CoV-2* and *Influenza A* and *B* genomes was consistent with previously reported ranges of 10–100 copies of RNA [24–34]. So, the sensitivity of our multiplex RT-qPCR assay is suitable for detection of *SARS-CoV-2* and *influenza A* and *B* in clinical specimens.

It should be noted, that we determined repeatability and reproducibility of the multiplex RT-qPCR assay using three different concentrations of each plasmid standard. The coefficients of variation of the intra-assay repeatability and inter-assay reproducibility of the multiplex RT-qPCR assay were < 5.0% and < 4.0% respectively, (Table 3), demonstrating that our multiplex RT-qPCR assay is an accurate and reliable diagnostic tool for detection of the *SARS-CoV-2*, *influenza A*, and *influenza B* viruses.

The multiplex RT-qPCR assay specifically amplified *SARS-CoV-2*, *influenza A*, and *influenza B* RNA, but did not amplify nucleic acids from other respiratory pathogens such as *respiratory syncytial virus (RSV), rhinovirus, Adenovirus, Epstein Barr virus*, and other *coronaviruses.*

For evaluation of the clinical performance, in total 70 samples were provided, including 44 positive and 26 negative samples that were confirmed by the COVID-19 reference laboratory in Golestan province without knowing their RT-qPCR results (blind). Out of these samples, the 10 new samples isolated from pharyngeal swab specimens during the sixth wave of the pandemic (Omicron) were used for virus detection by both our multiplex RT-qPCR and commercial multiplex kit. It is revealed that two samples out of 70 did not match with the results of the multiplex RT-qPCR assay and three samples did not match with the results of the commercial multiplex kit. Therefore, the diagnostic sensitivity of our multiplex RT-qPCR was higher compared to the commercial multiplex kit. It should be noted that two samples out of 70 were similarly detected as false-negatives by both assays. These controversial results in the detection rate of the viruses are dependent on many factors, including the low viral load in clinical samples, degradation of RNA genome or repeated freezing and thawing of samples, RT-qPCR inhibitors, and specificity of primers and probes.

The Cohen’s kappa coefficient revealed that there was a more agreement between our multiplex RT-qPCR and the singleplex RT-qPCR assay (as gold standard) in the detection of *SARS-CoV-2* (Table 5). In addition, the Passing-Bablok regression curves and Bland-Altman analysis between the Ct values of positive samples for *SARS-CoV-2*, *Influenza A*, and *Influenza B* revealed that there were high correlation between Ct values of our multiplex RT-qPCR and the commercial multiplex kit (Fig. 6). However, the average Ct value of the samples detected by our multiplex assay is slightly higher compared to the commercial multiplex kit. To overcome this undesired issue, we can improve the quality and robustness of our multiplex assay by improving the components of the reaction solution and the concentration of the primer-probe sets. Furthermore, our results showed that the multiplex assay is efficient for detecting all target genes (*N, M1, NS1* and *RNase* P) in the same reaction tube (Co-infected samples). Finally the comparison between the two multiplex assays shows that our assay has advantages over the commercial kit, mainly including less PCR running time and higher detection sensitivity.

A limitation in this study was that we did not have access to the more validated clinical sample. Furthermore, some studies used a similar number of clinical samples. It should be noted that expanding the sample amount minimizes technical errors [23, 33]. Another limitation is that the study was conducted on frozen respiratory samples, which can lead to decrease in the sample quality.

## Conclusion

In this study, a high-coverage multiplex RT-qPCR assay, with high analytical and diagnostic sensitivity and specificity was developed and validated. In a single reaction tube, it can facilitate the detection and differentiation of the *SARS-CoV-2* (*N* gene), *Influenza A* (*M1* gene), and *Influenza B* (*NS1* gene), thereby increasing the testing throughput and further reducing both cost and time significantly. This multiplex assay will be useful during *“Influenza* seasons” when *Influenza* is expected co-circulate with *SARS-CoV-2*.

### Electronic supplementary material

Below is the link to the electronic supplementary material.

Supplementary Material 1

## Data Availability

The authors confirm that all data generated or analyzed during this study are included in this published article [and/or] its supplementary information files.
