# Supplementary material for: Development of a robust TaqMan probe-based one-step multiplex RT-qPCR for simultaneous detection of SARS-CoV-2 and Influenza A/B viruses
Source: BMC Microbiol. 2023 Nov 11;23:335. doi: 10.1186/s12866-023-03048-9 (PMC10640757; doi:10.1186/s12866-023-03048-9)

**Supplemental Information**

**Supplemental Information 1.** Sanger sequencing of positive control plasmids (SARS-CoV-2-N, Influenza A-M1, and Influenza B-NS1 gene fragments)

**SARS-CoV-2-N gene contol plasmid (sequencing by M13/pUC sequencing forward primer):**

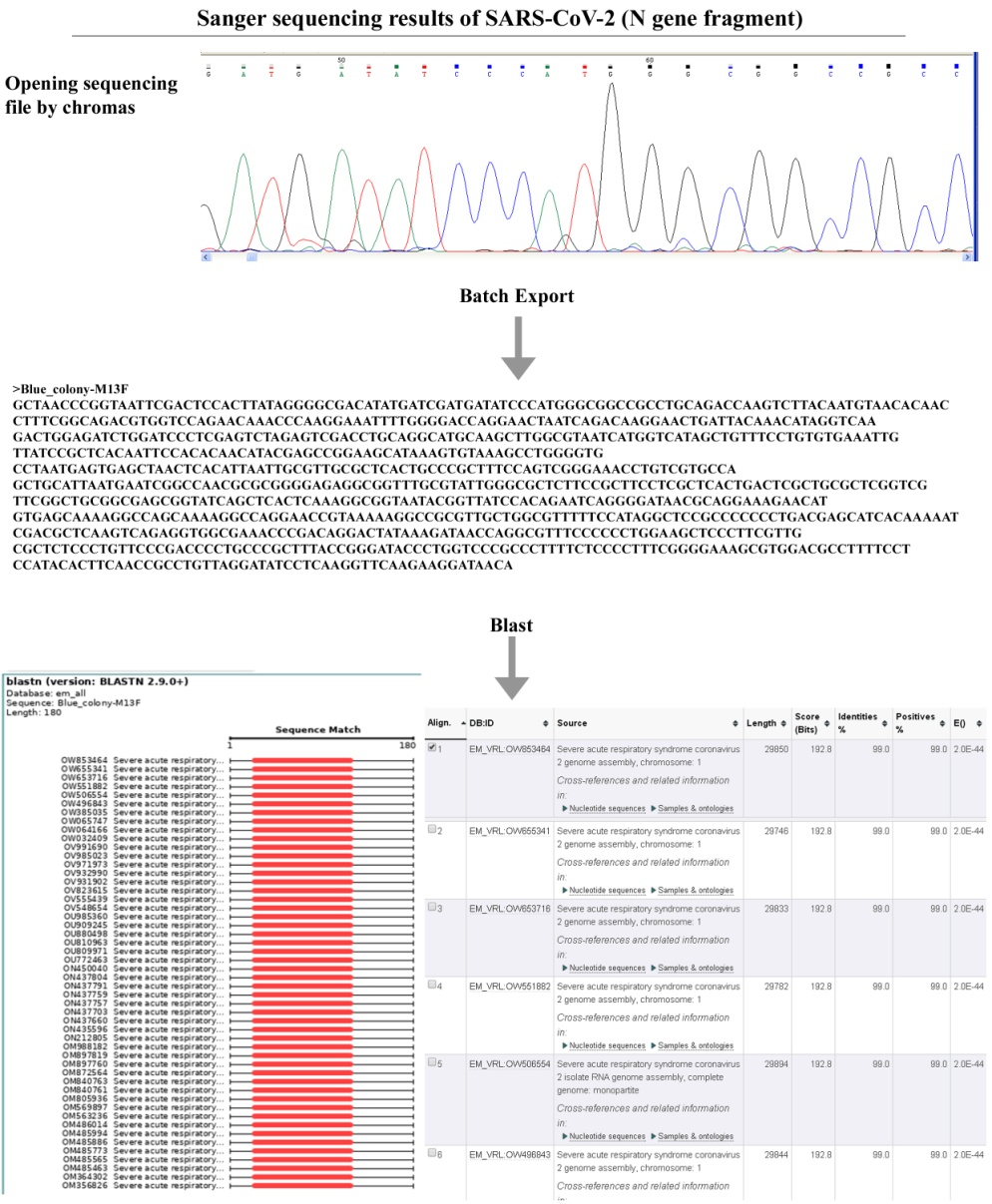


**Influenza A-M1 gene control plasmid (sequencing by influenza A forward primer):**

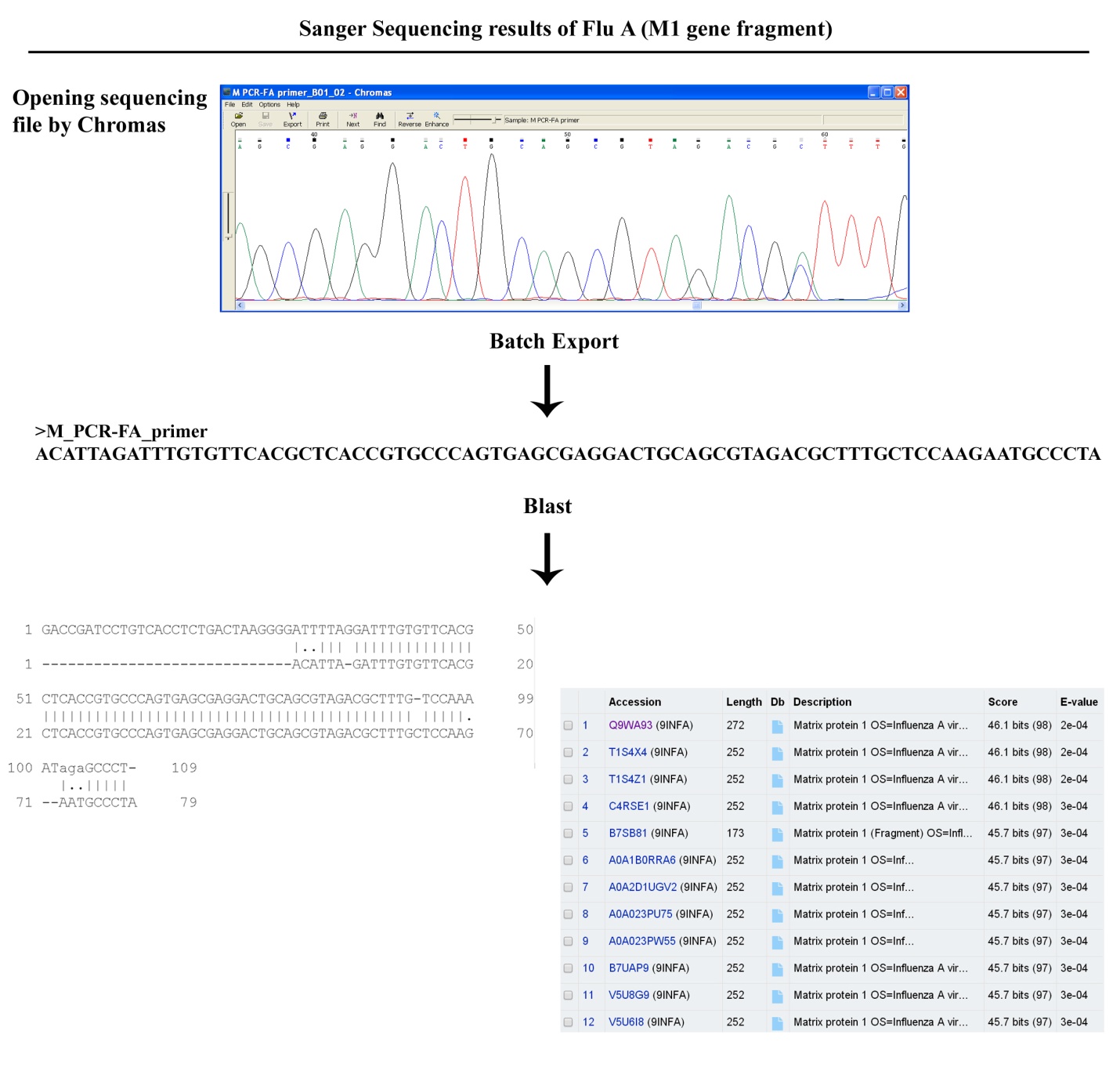


**Influenza B-NS1 gene control plasmid (sequencing by influenza B forward primer):**

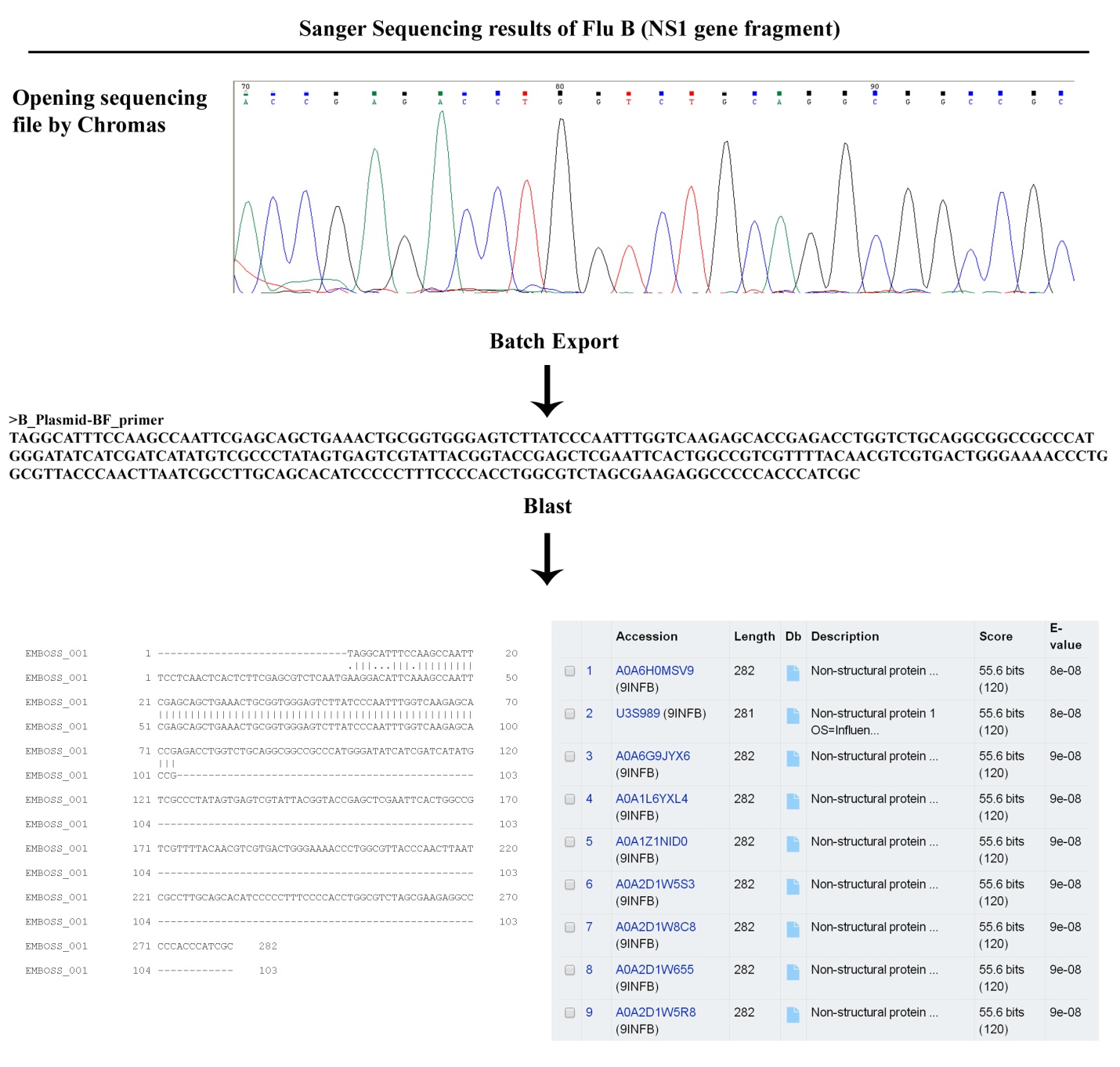


**Supplemental Information 2.** Alignment of SARS-CoV-2-specific Primer and probe sets with reference sequences for SARS-CoV-2, SARS-CoV, MERS, NL63, OC43, HKU1, and 229E. An in silico BLAST analysis of the primer and probe sequences for the SARS-CoV-2 confirmed that the assay is specific to SARS-CoV-2; it does not detect other human corona viruses.


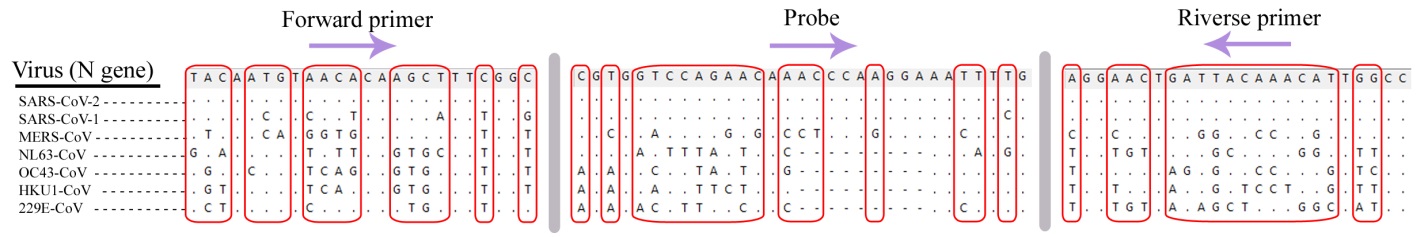


**Supplemental Information 3.** Optimization of primer sets using SYBR Green Real-time PCR. The electrophoresis and melting curve analysis of amplified *N* gene using conventional RT-PCR and SYBR Green Real-time PCR before creating mismatches and optimization (a). The obtained melting curves using SYBR Green Real-time PCR in simplex, duplex, triplex and qudruplex conditions with *SARS-CoV-2* and *Flu A/B* primers (b-f). The obtained standard curve and amplification plots using SYBR Green Real-time PCR with quantified *SARS-CoV-2* positive control plasmid (g). * The full length gels are shown below.


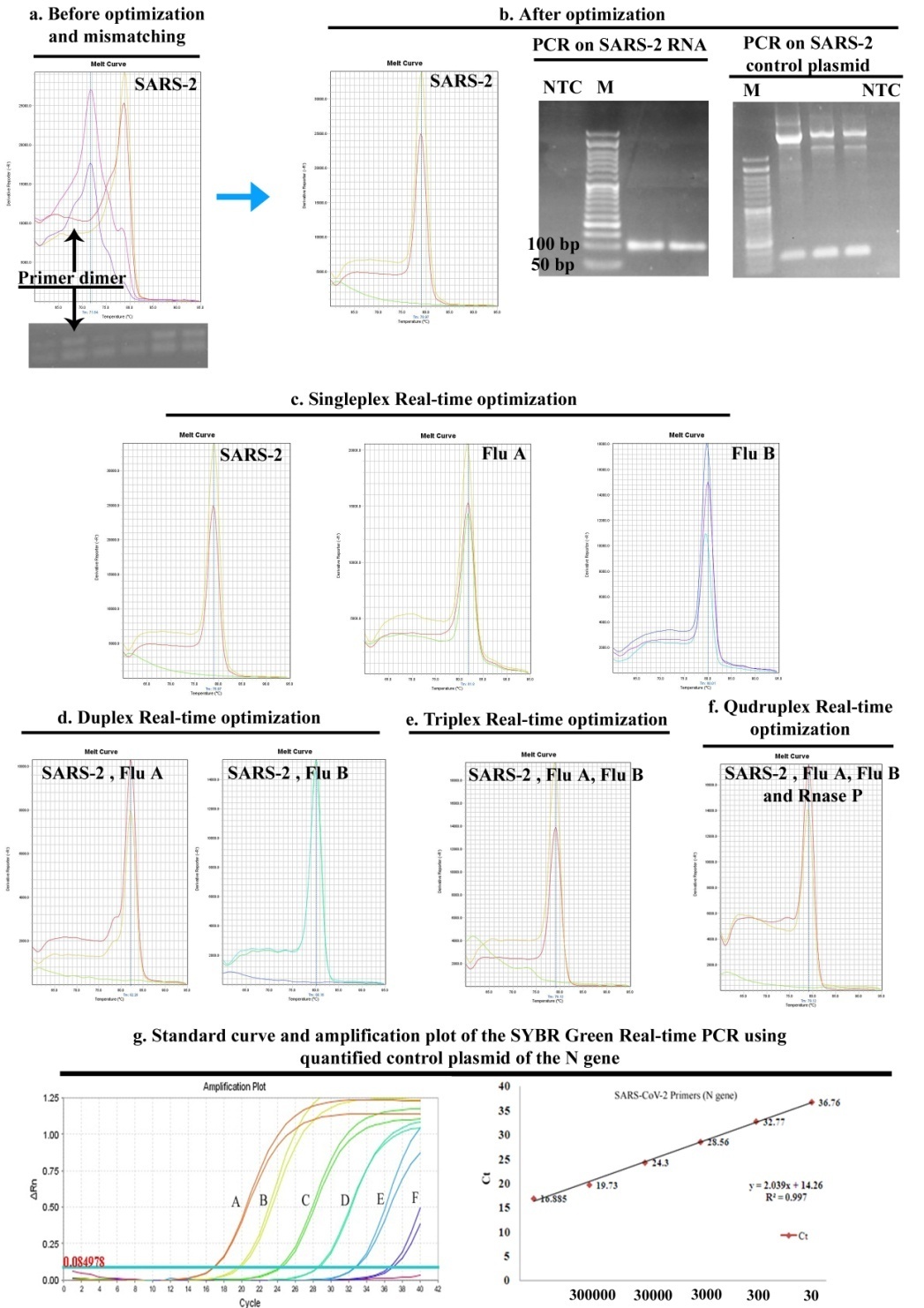


***Full length gels**

**
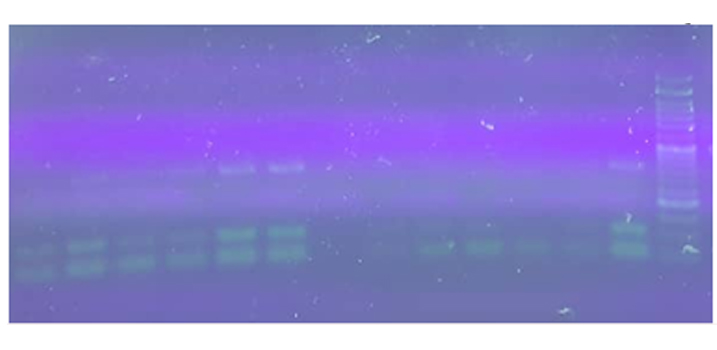
**

Before optimization of PCR reaction conditions and insertion of mismatches in primer-probe binding regions.

After optimization of PCR reaction conditions and insertion of mismatches in primer-probe binding regions.

**
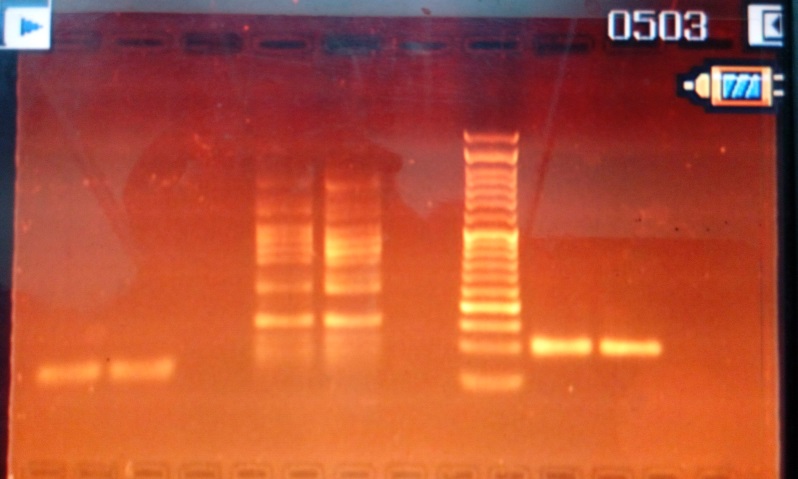
**

PCR on SARS-CoV-2 RNA

**
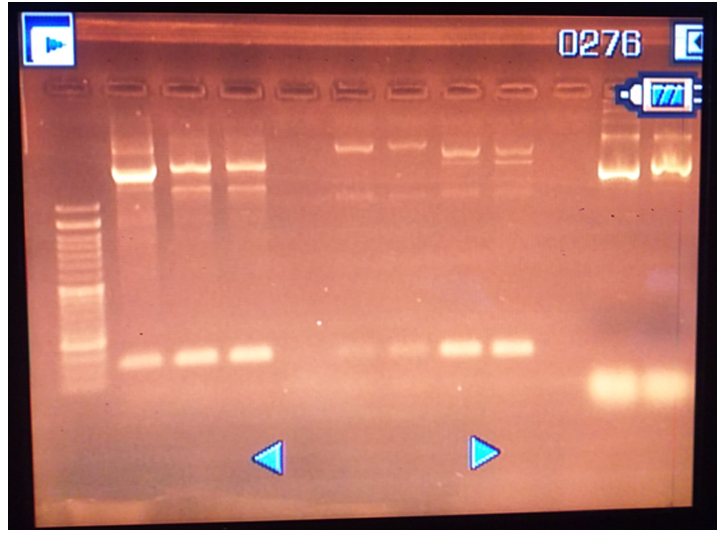
**

PCR on SARS-CoV-2 control plasmid

**Supplemental information 4.** Evaluation of analytical sensitivity of our multiplex assay using quantified standard plasmids

| Plasmid gene (Copies/reaction) | Cycle threshold (Ct) | | | | | | | | |
| --- | --- | --- | --- | --- | --- | --- | --- | --- | --- |
|  | SARS-CoV-2 | | | Influenza A | | | Influenza B | | |
| 300.000 | 23.32 | 23.22 | 22.94 | 20.91 | 20.96 | 20.77 | 20.31 | 20.18 | 19.39 |
| 30.000 | 25.93 | 25.93 | 26.03 | 23.63 | 23.90 | 23.91 | 23.24 | 23.99 | 22.68 |
| 3.000 | 30.09 | 29.92 | 29.53 | 27.74 | 27.72 | 27.79 | 26.15 | 25.03 | 26.59 |
| 300 | 33.01 | 32.48 | 32.88 | 31.63 | 31.73 | 31.80 | 29.99 | 30.35 | 29.72 |
| 30 | 34.66 | 35.26 | 34.61 | 33.93 | 34.27 | 34.12 | 33.49 | 32.83 | 33.95 |
| 3 | UD | UD | UD | 36.93 | 36.96 | 36.96 | 35.95 | 35.77 | 36.92 |
| 0.3 | UD | UD | UD | UD | UD | UD | UD | UD | UD |
| Negative control | UD | UD | UD | UD | UD | UD | UD | UD | UD |

UD, Undetected

**Supplemental information 5.** Summary of our multiplex assay specificity

| Virus strain | Our Multiplex Real-time RT-PCR (Ct value) | | | | | |
| --- | --- | --- | --- | --- | --- | --- |
|  | SARS-CoV-2 (N) | | Influenza A (M1) | | Influenza B (NS1) | |
| SARS-CoV-2 | 18.32 | 18.47 | UD | UD | UD | UD |
| IAV | UD | UD | 22.03 | 22.94 | UD | UD |
| IBV | UD | UD | UD | UD | 16.74 | 17.15 |
| Adenovirus B,C | UD | UD | UD | UD | UD | UD |
| Rhinovirus | UD | UD | UD | UD | UD | UD |
| Epstein-Barr virus (EBV) | UD | UD | UD | UD | UD | UD |
| Respiratory syncytial virus (RSV) A,B | UD | UD | UD | UD | UD | UD |
| Negative sample | UD | UD | UD | UD | UD | UD |
| Negative sample | UD | UD | UD | UD | UD | UD |
| Negative sample | UD | UD | UD | UD | UD | UD |

UD, Undetected

**Supplemental Information 6.** The obtained amplification plots for clinical samples by our multiplex assay


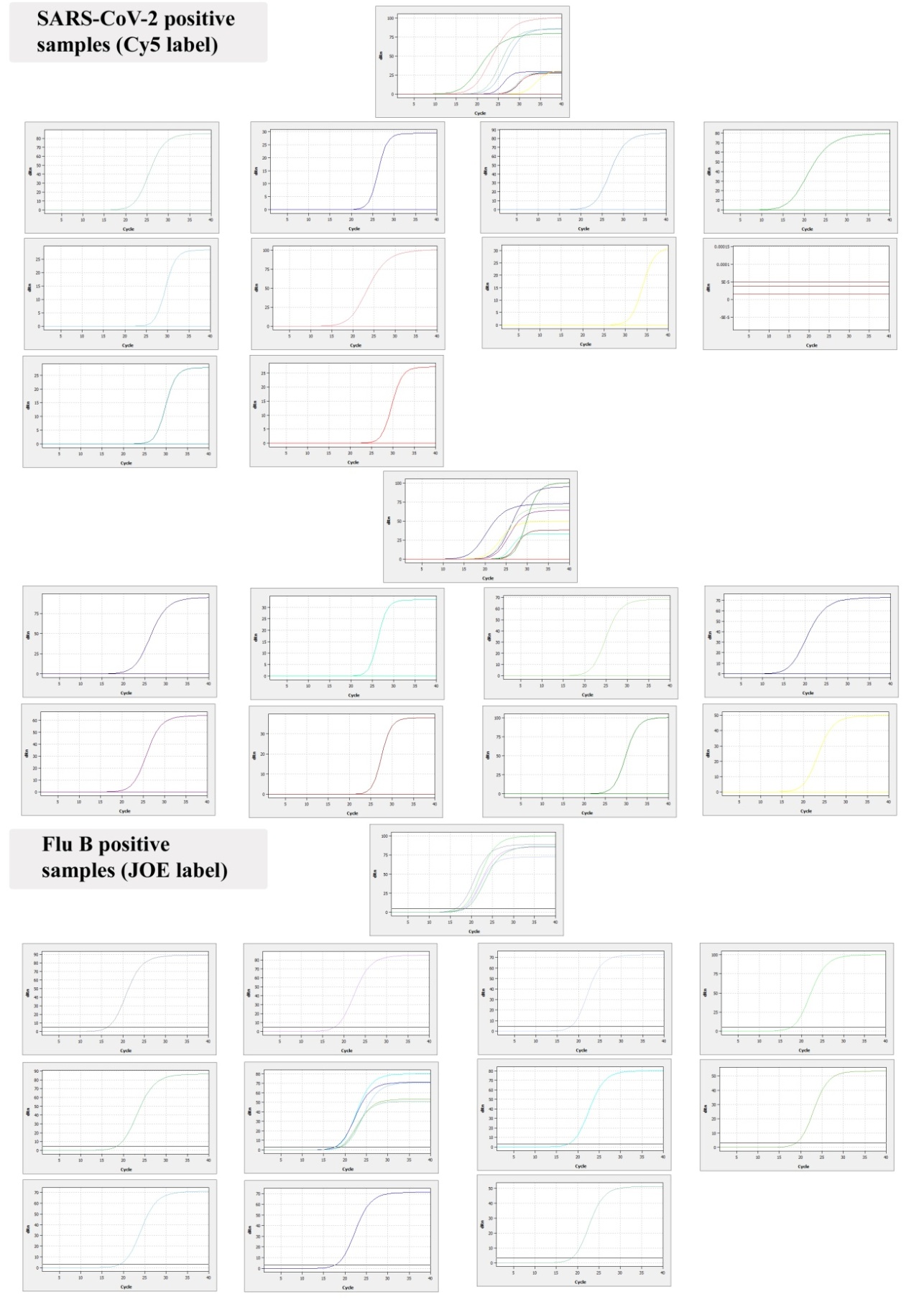


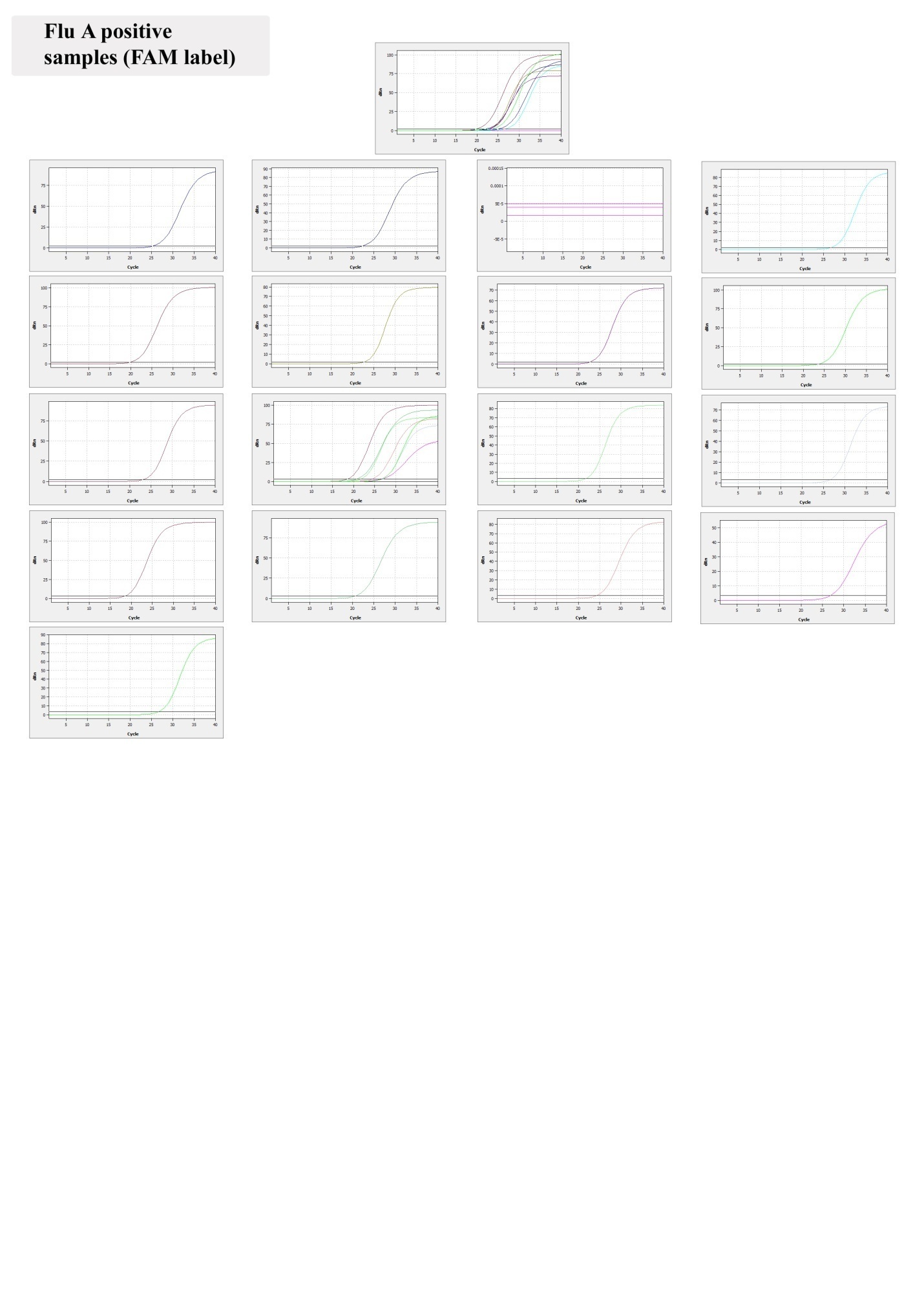


**Supplemental information 7.** The comparison of between various parameters of our multiplex assay and commercial multiplex kit.

| Variable | Our multiplex assay | Commercial kit |
| --- | --- | --- |
| Target genes (virus) | N, M1 and NS1 | N, M2 and NS1 |
| Internal control | RNase P | RNase P |
| Dyes | FAM, JOE, ROX, CY5 | FAM, Yakima Yellow, CY5, Texas Red |
| Instrument compatibility with dyes | QIAGEN, some BIORAD, Agilent, Cepheid, some ABI, Dropworks, Combinati, Roche, Stilla | QIAGEN, some BIORAD, Agilent, Cepheid, some ABI, Roche, Fluidigm |
| No. of amplification cycles | 40 | 45 |
| PCR running time | 85 min | 105 min |
| Cut off for positive (Ct) | 37 | - |
| Sample types | Nasopharyngeal | Nasopharyngeal |

**Supplemental information 8.** Testing co-infections

| Virus (target) | Assay | | | | | |
| --- | --- | --- | --- | --- | --- | --- |
|  | Our multiplex assay | | | Commercial kit | | |
|  | SARS-CoV-2 | IAV | IBV | SARS-CoV-2 | IAV | IBV |
| SARS-CoV-2, Influenza A/B | 19.21 | 19.41 | 16.86 | 19.65 | 19.24 | 16.31 |
| SARS-CoV-2, Influenza A/B | 19.77 | 22.3 | 17.48 | 19.26 | 19.78 | 16.05 |
| SARS-CoV-2, Influenza A/B | 20.61 | 20.45 | 17.31 | 19.87 | 18.18 | 16.39 |
| SARS-CoV-2, Influenza A/B | 22.09 | 23.23 | 18.1 | 16.48 | 18.13 | 18.49 |
| SARS-CoV-2, Influenza A/B | 21.13 | 22.24 | 20.81 | 21.74 | 20.12 | 18.93 |
| SARS-CoV-2, Influenza A/B | 24.52 | 20.38 | 20.87 | 24.13 | 23.31 | 19.22 |
| SARS-CoV-2, Influenza A/B | 29.85 | 24.53 | 20.87 | 25.54 | 25.58 | 19.71 |
| SARS-CoV-2, Influenza A/B | 18.82 | 19.41 | 20.02 | 20.56 | 26.61 | 18.66 |
| Ct average | 22.00 | 21.49 | 19.04 | 20.90 | 21.36 | 17.97 |

IAV, Influenza A Virus; IBV, Influenza B Virus

**Supplemental Information 9.** The amplification plots of detected co-infected samples by our multiplex assay an


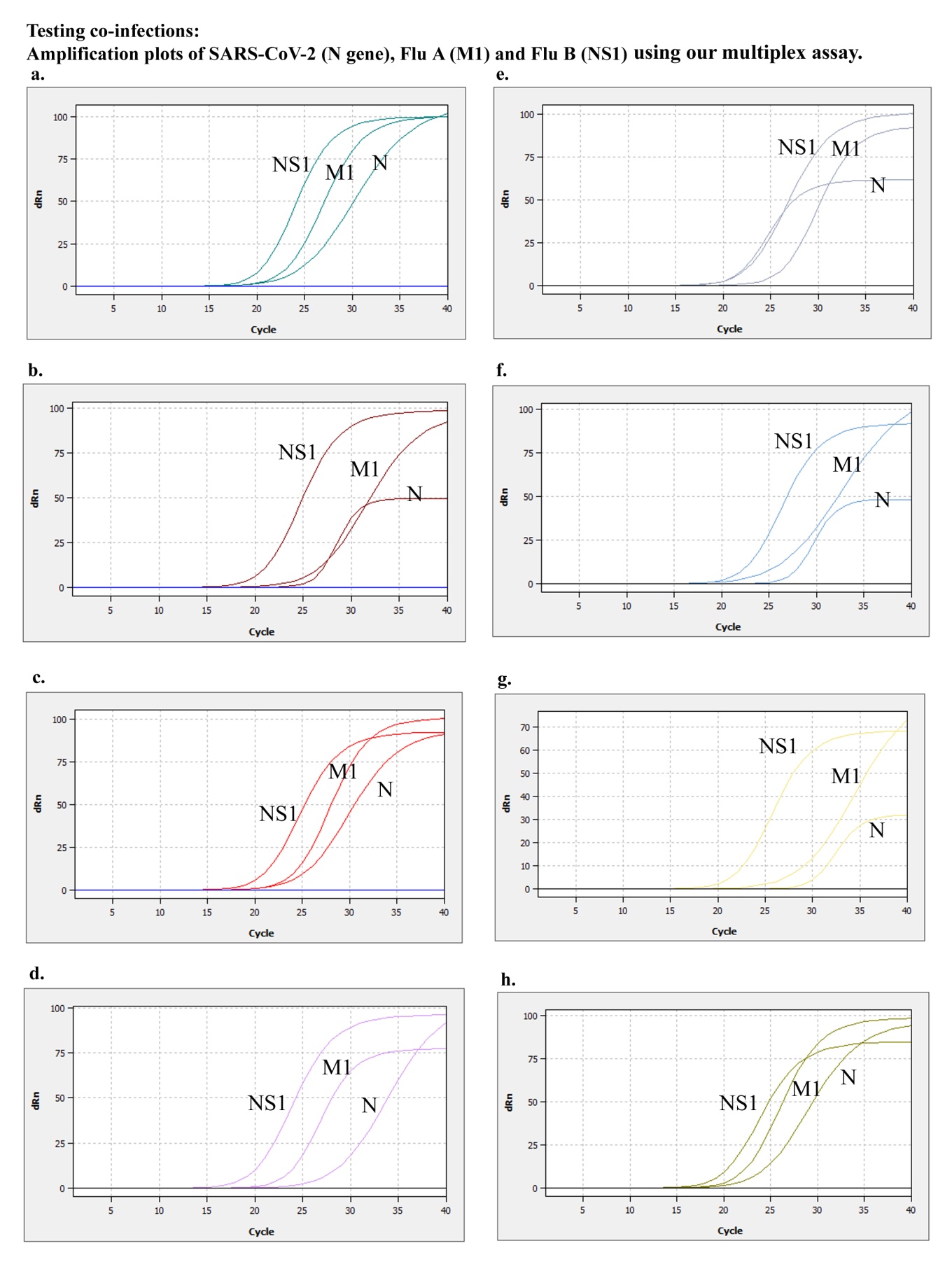

Supplement: Supplementary file 1 — Supplementary Material 1 [file 12866_2023_3048_MOESM1_ESM.docx]
